# Angiotensin 1-7: A Novel Strategy in COVID-19 Treatment

**DOI:** 10.34172/apb.2020.068

**Published:** 2020-08-09

**Authors:** Hamed Imanpour, Haleh Rezaee, Masoud Nouri-Vaskeh

**Affiliations:** ^1^Tabriz University of Medical Sciences, Tabriz, Iran.; ^2^Infectious Diseases and Tropical Medicine Research Center, Tabriz University of Medical Sciences, Tabriz, Iran.; ^3^Department of Clinical Pharmacy (Pharmacotherapy), Faculty of Pharmacy, Tabriz University of Medical Sciences, Tabriz, Iran.; ^4^Immunology Research Center, Tabriz University of Medical Sciences, Tabriz, Iran.; ^5^Network of Immunity in Infection, Malignancy and Autoimmunity (NIIMA), Universal Scientific Education and Research Network (USERN), Tehran, Iran.

## Dear Editor,

Coronavirus disease 2019 (COVID-19) is an emerging infection caused by severe acute respiratory syndrome coronavirus 2 (SARS-CoV-2). The mortality rate of COVID-19 is high and there is no effective treatment for these patients to reduce the high hospitalization and mortality rates.

The renin-angiotensin-aldosterone system plays a critical role in COVID-19 pathogenesis.^1^ The sex difference in the mortality rate and complications of COVID-19, and also the more favorable prognosis of children leads to new hypotheses regarding the protective and harmful factors in the treatment of these patients.^2,3^

Angiotensin-converting enzyme (ACE) plays a role in innate and adaptive immune responses as well as converting angiotensin and affecting different physiological functions.^4^ Understanding the expression of ACE on myeloid cells can be helpful in the treatment of infections. In comparison to adults, children have a higher level of ACE.^4^ Although SARS-CoV-2 binds to ACE2 for entering the host cells, children are more immune against this virus; this is possibly due to a high level of ACE in children and its effects on immune responses.^4^ Moreover, although children have a higher level of renin, angiotensin, and aldosterone compared with adults and also a higher amount of fluid in their bodies, they have lower blood pressures^5^; one of the reasons behind this is the high level of angiotensin 1-7 that acts as a vasodilator and anti-inflammatory agent against angiotensin 2. This is probably another reason for children’s enhanced immunity against COVID-19.^6^ Despite the low level of ACE2 in females in comparison to males, which protects the host against virus penetration, given the regulatory effect of estrogen on angiotensin type 2 receptor its effect on angiotensin 1-7 is more dominant. This is possibly the relative cause of women’s increased immunity against COVID-19 infection.^7^ Angiotensin receptor blockers (ARBs) are one of the main drugs that can provide high levels of ACE and angiotensin 1-7 at the same time. However, ARBs may also elevate the ACE2 level as the virus entry location, which should be considered in the prescription of ARBs.^8,9^

The administration of angiotensin 1-7 in adults may provide immunity against COVID-19 as in children. By injecting angiotensin 1-7, the renin-angiotensin-aldosterone axis will become active to prevent a further drop in blood pressure, the ACE level will rise, and the ACE2 level will reduce owing to the accumulation of angiotensin 1-7.^8^ This means that providing high levels of angiotensin 1-7 and ACE while reducing inflammatory bradykinin will be protective against ACE2, the entry site of the virus into the host cells.^8^ Finally, the controlled injection of angiotensin 1-7 as a modulator of the renin-angiotensin-aldosterone system and the compensation of a possible drop in blood pressure by infusion of intravenous fluids and alpha agonists may be able to reduce the severity of COVID-19 infection since the host is given an opportunity to induce specific immunity.

## Ethical Issues

Not applicable.

## Conflict of Interest

We declare no competing interests.

## References

[1] Busse LW, Chow JH, McCurdy MT, Khanna AK (2020). COVID-19 and the raas—a potential role for angiotensin II?. Crit Care.

[2] Wenham C, Smith J, Morgan R (2020). COVID-19: The gendered impacts of the outbreak. Lancet.

[3] Balasubramanian S, Rao N, Goenka A, Roderick M, Ramanan A (2020). Coronavirus disease (COVID-19) in children - what we know so far and what we do not?. Indian Pediatr.

[4] Bernstein KE, Khan Z, Giani JF, Cao DY, Bernstein EA, Shen XZ (2018). Angiotensin-converting enzyme in innate and adaptive immunity. Nat Rev Nephrol.

[5] Broughton Pipkin F, Smales OR, O’Callaghan M (1981). Renin and angiotensin levels in children. Arch Dis Child.

[6] Pinheiro SV, Simões E Silva AC (2012). Angiotensin converting enzyme 2, Angiotensin-(1-7), and receptor MAS axis in the kidney. Int J Hypertens.

[7] Sullivan JC, Rodriguez-Miguelez P, Zimmerman MA, Harris RA (2015). Differences in angiotensin (1-7) between men and women. Am J Physiol Heart Circ Physiol.

[8] Rossi GP, Sanga V, Barton M (2020). Potential harmful effects of discontinuing ACE-inhibitors and ARBs in COVID-19 patients. Elife.

[9] Mourad JJ, Levy BI (2020). Interaction between raas inhibitors and ace2 in the context of covid-19. Nat Rev Cardiol.

